# *In Vitro* and *In Vivo* Investigation of the Potential of Amorphous Microporous Silica as a Protein Delivery Vehicle

**DOI:** 10.1155/2013/306418

**Published:** 2013-08-07

**Authors:** Amol Chaudhari, Lieve Vanmellaert, Matthias Bauwens, Peter Vermaelen, Christophe M. Deroose, Ignace Naert, Marcio Vivan Cardoso, Johan A. Martens, Joke Duyck

**Affiliations:** ^1^Department of Prosthetic Dentistry, BIOMAT Research Group, KU Leuven, Kapucijnenvoer 7, 3000 Leuven, Belgium; ^2^Laboratory of Bacteriology, Rega Institute, KU Leuven, Minderbroedersstraat 10, 3000 Leuven, Belgium; ^3^Laboratory of Radiopharmacy, KU Leuven, Herestraat 49, 3000 Leuven, Belgium; ^4^Nuclear Medicine and Molecular Imaging, KU Leuven, Herestraat 49, 3000 Leuven, Belgium; ^5^Centre for Surface Chemistry and Catalysis, KU Leuven, Kasteelpark Arenberg 23, 3001 Heverlee, Belgium

## Abstract

Delivering growth factors (GFs) at bone/implant interface needs to be optimized to achieve faster osseointegration. Amorphous microporous silica (AMS) has a potential to be used as a carrier and delivery platform for GFs. In this work, adsorption (loading) and release (delivery) mechanism of a model protein, bovine serum albumin (BSA), from AMS was investigated *in vitro* as well as *in vivo*. In general, strong BSA adsorption to AMS was observed. The interaction was stronger at lower pH owing to favorable electrostatic interaction. *In vitro* evaluation of BSA release revealed a peculiar release profile, involving a burst release followed by a 6 h period without appreciable BSA release and a further slower release later. Experimental data supporting this observation are discussed. Apart from understanding protein/biomaterial (BSA/AMS) interaction, determination of *in vivo* protein release is an essential aspect of the evaluation of a protein delivery system. In this regard micropositron emission tomography (**μ**-PET) was used in an exploratory experiment to determine *in vivo* BSA release profile from AMS. Results suggest stronger *in vivo* retention of BSA when adsorbed on AMS. This study highlights the possible use of AMS as a controlled protein delivery platform which may facilitate osseointegration.

## 1. Introduction

 Protein adsorption plays a key role in many natural processes such as transmembrane signaling or blood coagulation cascade [1], vascularization around artificial tissue scaffolds [2], and interaction of biomedical implants with blood stream proteins that eventually leads to implant integration in the body [3]. Knowledge of protein interaction with biomedical implants can also be exploited for the development of implant systems with a certain desired effect. Understanding the role of protein molecules at the implant surface is essential in searching for controlling early events during the process of implant osseointegration [4]. Bone induction starts with the stimulation of undifferentiated and pluripotent cells in the vicinity of an implant into the bone forming cell lineage [5]. During these early events and later, different growth factors (GFs) are released. In case of oral and orthopedic implants, GFs are known to affect different phases of the osteoblast differentiation lineage [6, 7]. In the past few decades researchers have been investigating bone morphogenetic proteins (BMPs) [8] which are known to induce bone regeneration [9, 10]. 

 There is a general consensus that, to maximize their osteoinductive efficacy, BMPs need to be carried and delivered in a controlled and sustained manner rather than in a burst [11–13]. These approaches are aimed at delivering the BMPs in free form at an appropriate location until the bone is sufficiently healed for functional loading. Most of these approaches are based on diffusion- or erosion-controlled delivery of BMPs either incorporated or immobilized on the implant surface with or without the help of biocompatible coatings. Many of the BMP delivery systems [12, 14–20] that are being investigated have some shortcomings. Low mechanical strength and uncontrolled degradation (ceramic materials) [21], immunogenicity, difficulty in processing and a potential risk of transmitting animal-originated pathogens (natural polymers) [22], acute and/or chronic inflammatory response, and potential localized pH shifts which may also be important for functioning of incorporated or immobilized BMPs (synthetic polymers) [23] are few disadvantages. Moreover, information regarding the necessary amount of BMPs and related release kinetics from the biomaterial to be used clinically as a delivery vehicle for particular type of tissue regeneration is unknown to a large extent [24] and expected to be varying depending on the implant size, site of application, type of bone, and so forth. To overcome these issues, it is necessary to find new delivery systems which are either inert to the bone formation process or support it with a possibility of changing the protein release kinetics without affecting the protein's biological function.

 In this study the potential of amorphous microporous silica (AMS) as a protein delivery vehicle was evaluated. AMS is a material with uniform pore size similar to zeolites. Note that the term “microporous” may be misleading. But pores smaller than 2 nm have been classified as micropores by IUPAC [25]. AMS has already shown its potential as a carrier and delivery vehicle for small drug molecules [26] and antiseptic molecules [27]. Although AMS pores are too narrow for accommodating proteins, the latter can potentially be adsorbed on the external surface of AMS particles or coatings. Moreover, a smaller molecule loaded inside the micropores can be delivered separately or simultaneously by diffusion out of the pores having another function, such as biofilm inhibition [27]. Synthesis of AMS is a simple procedure, and it can be used either in the powder form or as coating [26, 27]. AMS is a biocompatible [28] and hydrophilic [26] material which may be an advantage for its intended use as a BMP delivery vehicle aiming for faster implant osseointegration. Moreover, it does not display adverse effects on the survival and proliferation of human mesenchymal stem cells [29], and the bone regeneration around AMS-coated titanium implants has been reported to be similar to noncoated titanium implants [30].

 Apart from designing implant surfaces for carrying and effectively releasing BMP molecules for faster osseointegration, determining BMP release profiles from proposed materials *in vivo* is another challenge. In this regard radiolabeling of the protein molecules can be used to evaluate protein release/retention *in vivo* [31, 32]. Micropositron emission tomography (*μ*-PET) is one technique which can be used to monitor the protein molecules and their release/retention *in vivo* at the implant site. *μ*-PET is a noninvasive technique to measure the concentration of positron emitting isotopes in living entities [33]. With this ability it can be used to measure the concentration of a variety of molecules *in vivo* such as receptors, antigens, enzymes, and reporter gene products, [33], and therefore it can be used to study specific aspects of metabolism [34] and immunology [35].

The aim of this work was to study the potential of AMS as protein carrier and delivery vehicle. For this study, bovine serum albumin (BSA) was used as a model protein. It was hypothesized that the micropores of AMS will increase the protein/surface interaction by providing larger surface area for the protein molecules and thereby increasing the retention and availability of the protein for release. Adsorption of BSA on AMS was explored in order to study protein loading capacity of AMS and BSA release profile was determined in aqueous environment. Besides the *in vitro* evaluation, the potential of *μ*-PET for *in vivo* monitoring of proteins in a bone cavity was also studied. In this explorative experiment, effect of adsorption of bovine serum albumin (BSA) on amorphous microporous silica (AMS) on the retention of the former at a specific site in an animal was evaluated as well using *μ*-PET.

## 2. Materials and Methods

### 2.1. AMS Synthesis

 AMS was synthesized as documented previously [36]. Tetraethyl orthosilicate (TEOS; Acros, Germany; 98%), technical ethanol (VWR, Belgium), and HCl (ChemLab, Belgium; 37%) were combined maintaining a molar ratio of TEOS : ethanol : HCl : water as 1 : 3 : 1.74 : 6. The solution was stirred for 24 h. The sol was subsequently heated at 50°C for 3 days. Then, the gel body was broken and heated up to 65°C at a heating rate of 0.1°C/min. The temperature was maintained for 5 h and then heated further to 300°C at a heating rate of 0.1°C/min. The temperature was kept constant for 5 h and then cooled to room temperature. AMS xerogel was crushed first with mortar and pestle and then by a ball mill at a speed of 500 rpm for 1 h. This limited speed of milling process was used to prevent structural damage and loss of porosity [37]. For *in vivo* experiment, AMS particles, crushed with mortar and pestle, were sieved, and the fraction of particles between the size of 80 *μ*m and 125 *μ*m was chosen for the experiment.

### 2.2. Protein Loading

 Sodium phosphate buffer (PB) solutions with an ionic strength of 10 mM and required pH were prepared by mixing appropriate volumes of 10 mM NaH_2_PO_4_ (Fluka, Germany, ≥99%) and 10 mM Na_2_HPO_4_ (Sigma, Germany, ≥98.5%). BSA (Sigma, Germany, A7030, 98%) solutions were prepared in PB at pH 5.5 and 7.0. For the determination of BSA adsorption isotherms, BSA was adsorbed on 100 mg AMS from 5 mL solution with bulk protein concentrations in the range of 0.4 to 10 mg/mL. The adsorption was carried out by continuous end-over-end shaking of the mixture at 50 rpm for 1 h at room temperature. Shaking was just enough to keep AMS particles in suspension to obtain reproducible adsorption. After adsorption, the suspensions were centrifuged at 4000 rpm for 5 min, and the supernatant was carefully removed. Both components after separation were stored at 4°C until further analysis.

 For the release experiments, BSA was adsorbed on 100 mg AMS via 5 mL of solution in PB (pH 5.5; 10 mM) having a bulk concentration of 10 mg/mL. This procedure ensured that AMS had an equilibrium BSA concentration. The equilibrium BSA concentration corresponds to the maximum amount of protein adsorbed on AMS surface as determined by adsorption isotherm. Initial bulk BSA concentration (10 mg/mL) used for the preparation of samples for release experiments was decided from the results of BSA adsorption isotherm. The adsorption and release experiments were repeated, and two sets of samples were analyzed to determine the adsorption isotherm and release profiles. 

### 2.3. Protein Release

 AMS with adsorbed BSA with equilibrium protein concentration was mixed with 5 mL fresh PB (pH 5.5; 10 mM) at room temperature. The amount of BSA released was determined at regular intervals (10 min, 30 min, 1 h, 2 h, 6 h, 12 h, and 24 h). For each release time point, a separate AMS batch with adsorbed protein was used. Physical and environmental stimuli (end-over-end shaking, pH, and buffer concentration) were similar for both the protein adsorption and release experiments. At each release time point the mixtures were centrifuged at 4000 rpm for 5 min, and supernatant was removed and stored at 4°C until BSA quantification. AMS after BSA release was dried at 37°C for further surface analysis.

### 2.4. Protein Quantification for the Evaluation of *In Vitro* Protein Adsorption and Release

 BSA quantification was carried out by Bradford assay [38]. BSA solutions in PB with concentrations in the range 2 to 25 *μ*g/mL were prepared for plotting a standard curve. 0.8 mL protein solution and 0.2 mL Bradford reagent (BioRad, Germany) were mixed thoroughly and kept in a dark place for 5 min. Absorbance at 595 nm was measured using a plate reader (Infinite F200, TECAN, Austria), and a standard curve was determined. A series of dilutions of BSA solutions obtained after both the adsorption, and release experiments was prepared and the amount of BSA was determined using the standard curve. For quantification of BSA in solutions, dilutions were taken into consideration for solutions with protein concentration higher than the standard curve range.

 The amount of BSA adsorbed on AMS was determined using bulk protein concentration and the equilibrium protein concentration after adsorption experiment. Adsorption isotherms were determined by plotting the amount of protein adsorbed on AMS versus equilibrium protein concentration. BSA release profile from AMS was determined by plotting the amount of BSA released (in %) as a function of time.

### 2.5. Nitrogen (N_2_) Physisorption Isotherms

 N_2_ physisorption experiments were carried out to characterize surface area of AMS as synthesized, after protein loading and after protein release in order to monitor changes in the porous structure due to adsorption and release experiments. AMS without adsorbed BSA, AMS with maximal adsorbed BSA and AMS after BSA release experiments at 3 different release times of 10 min, 6 h, and 24 h were characterized. AMS with adsorbed BSA was calcined at 400°C in air for 24 h to eliminate the protein present on the surface. N_2_ adsorption/desorption isotherms were recorded at −196°C (Tristar3000, Micromeritics, USA). Before the measurements, all the samples were dried for 12 h at 250°C under N_2_ flow (SmartPrep, Micromeritics, USA). Specific surface area and micropore volume were determined using BET [39] and *t*-plot [40] analysis. 

### 2.6. Scanning Electron Microscopy (SEM)

AMS particles before and after adsorption of BSA were qualitatively analyzed using a field-emission-gun scanning electron microscope (Feg-SEM, XL 30, Phillips, the Netherlands). For SEM, the particles were fixed on metal surface and coated with a gold layer by sputtering.

### 2.7. Zeta (*ζ*) Potential Measurement

 Charged colloidal particles such as protein molecules and AMS particles in PB are surrounded by a double layer of counter ions. The *ζ* potential is an electrokinetic potential of a colloidal particle [41]. *ζ* potential of AMS and BSA was determined under experimental conditions of protein adsorption using BIC ZetaPALS (Brookhaven Instruments Corporation, USA). For AMS, a small amount of particulate AMS was mixed with 1.5 mL PB so that the particles remained in suspension during the measurement.

### 2.8. Radioiodination of BSA and Adsorption on AMS

Radioiodination of BSA (Sigma, Germany) was performed by carrier-added electrophilic substitution with IODO-GEN (Pierce, Europe) as an oxidizing agent. In short, [^124^I]-NaI (IBA, Belgium; half-life 4.18 days) is transferred to an IODO-GEN vial and mixed with 100 *μ*L phosphate buffer (pH 7.5, 100 mM). Subsequently, 100 *μ*L of 0.1% (w/v) BSA solution in water is added, and the reaction is allowed to proceed at room temperature for 15 minutes, while shaking gently. Purification was performed by filtering (10 kDa MWCO filter unit, 1.5 mL capacity, Sigma, Germany) the solution under centrifugation for 5 min at 12000 g. After washing with 0.5 mL saline solution and centrifugation, 40 *μ*L of purified ^124^I-labeled BSA (^124^I-BSA) solution was recovered. A part of the solution was analyzed by HPLC, using a BioSep-SEC-S 3000 column (Phenomenex, the Netherlands) with 10 mM phosphate buffer (pH 7) as a mobile phase at a flow rate of 1 mL/min. Radiochemical purity of at least 99% was obtained. Overall yield was more than 90% with losses mainly due to transfers and stickiness to the centrifuge filter unit. Remaining solution was further diluted to a final volume of 400 *μ*L in saline solution. For mixing with AMS, 100 *μ*L of radiolabeled protein was mixed with 10 mg of AMS particles in a small vial where the protein was allowed to adsorb on the substrate for 1 h.

### 2.9. Surgical Procedure for the *In Vivo* Protein Release Analysis

**Figure 1:**
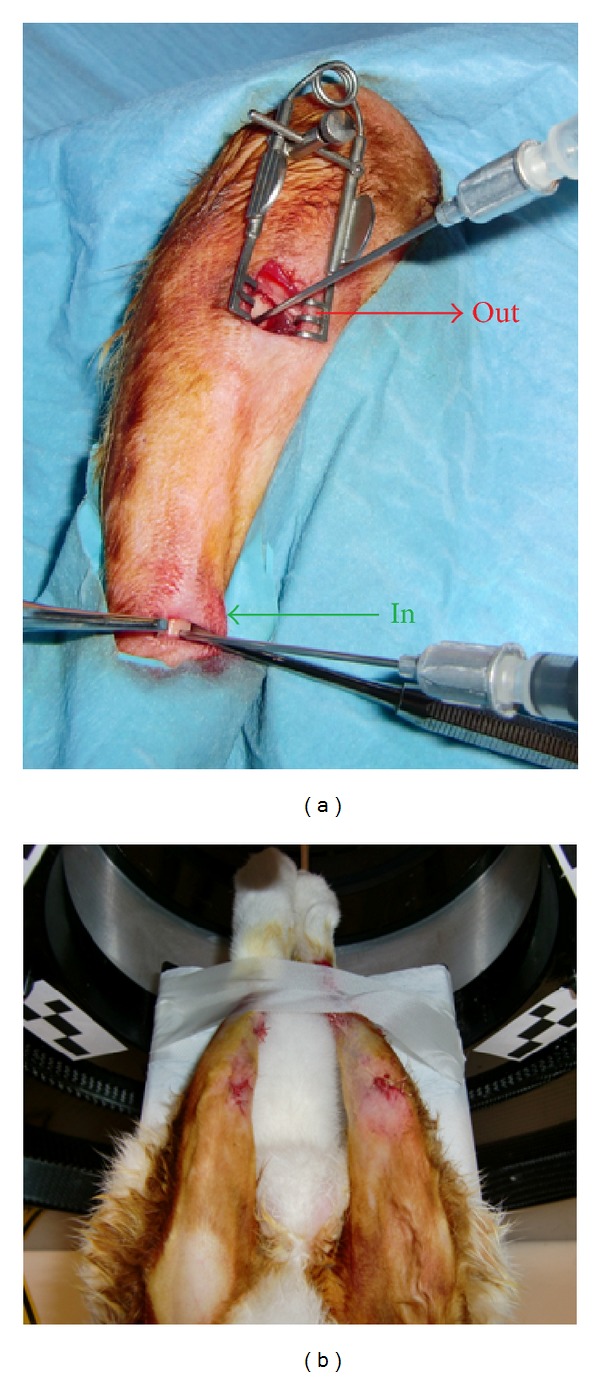
Surgical procedure, *μ*-PET scanning, and image analysis. (a) Two holes were drilled in each tibia: one for injecting the protein solution (IN) with (test) or without (control) AMS and the second one for observation of any possible overflow (OUT). Both test and control materials were injected at same time. The holes were sealed using elastomeric material and the incisions were sutured. (b) After suturing the first *μ*-PET, scanning was started immediately.

The research protocol was approved by ethical committee for laboratory animal research of KU Leuven and was performed according to Belgian animal welfare regulations and guidelines (approval ID: P142/2009). ^124^I-BSA adsorbed on AMS [AMS-(^124^I-BSA)] was considered as the test condition whereas ^124^I-BSA was used as the control. Both materials were inserted in the bone marrow cavity of each tibia of a 6-month-old New Zealand white rabbit. The animal was kept in quarantine for 4 weeks before the surgery. Surgery was performed under aseptic conditions. First, the animal was premedicated with an intramuscular neuroleptic analgesic (Vexylan, 1 mg/kg, CEVA, Belgium) and intramuscular anesthetic (Ketamine 1000 15 mg/kg, CEVA, Belgium). During the surgery, anesthesia was maintained using Isoflurane (Halocarbon, USA). After applying local anesthetic (Lignospan, Septodond, France) subcutaneously at a medial side of proximal tibia, two small longitudinal incisions were made approximately at both proximal and distal ends of the bone marrow cavity (Figure 1(a)) of each tibia, and both soft tissues and periosteum were mobilized. A hole was made at each incision with a low-speed hand piece and a surgical drill (Ø 1 mm) under copious saline cooling. Cortical bone was perforated till the bone marrow cavity. The size of the hole was exactly 1 mm for a blunt needle tip (Kendall, USA) to be press-fitted. 100 *μ*L each of test [AMS-(^124^I-BSA)] and control (^124^I-BSA) materials was drawn into the syringes, and the syringes were then fixed to the tips already in position in the distal holes while 2 empty syringes were fitted in the remaining proximal holes. The contents of both the syringes containing the test and the control materials were injected simultaneously and completely into the bone marrow cavity of the tibia (Figure 1(b), “IN”). The remaining two syringes were observed for any outflow (Figure 1(a), “OUT”), but no liquid was observed in these syringes. The holes in the bone were then sealed with elastomeric material (Permadyne base paste and Permadyne catalyst paste, 3 M ESPE, Germany), and the skin was sutured. Postoperatively, the animals were medicated with intramuscular buprenorfin as analgesic (Temgesic 0.05 mg/kg body weight, Schering-Plough NV, Belgium) and intramuscular antibiotic (penicillin) for 3 days at a dose of 300.000 IU/day (Kela NV, Belgium). After 4 days, the animals were sacrificed with a 0.1 mL/kg body weight intravenous injection of an embutramide-mebenzoniumjodide-tetracaine HCl solution (T61, Intervet, Belgium).

### 2.10. *μ*-PET Scanning and Image Analysis

Dynamic *μ*-PET imaging without attenuation correction was performed on a Focus 220 device (Concorde Microsystems Inc., USA) (Figure 1(b)). The first scan was dynamically recorded for 1 h (12 frames of 5 minutes each) starting immediately after completion of surgical procedure. A continuous bed motion covering the complete tibia was employed, and each frame corresponded to five minutes. Further scans were recorded at 4, 24, 48, and 96 h (15 min each with bed motion of 5 min; all data summed to one image). For these scans the animal was sedated by intramuscularly administered neuroleptic analgesic and anesthetic in the similar manner as used for surgical procedure. Quantitative image analysis was performed with PMOD software (version 3.1.; PMOD Inc., Switzerland). Regions-of-interest (ROI) were drawn around the activity site in both legs of the animal in each slice where the activity was observed. The total activity in each tibia was determined at each time point by averaging the measured activity of each slice of the image. The activity at the first time point (0 h) was considered as the amount of activity injected and the decay-corrected activities at subsequent time points were normalized to this initial activity. ^124^I-BSA retention in terms of retention of total activity in each tibia was plotted as a function of time.

## 3. Statistical Analysis

 Statistical analysis for comparing *in vivo* BSA retention profiles was carried out by SPSS software (SPSS 13.0, SPSS Inc., USA). The data distribution was normal as confirmed by Shapiro-Wilk test and Q-Q plot. A paired *t*-test was carried out to find out differences (*P* < 0.05) in the protein retention profiles.

## 4. Results

### 4.1. Protein Adsorption Isotherms

**Figure 2:**
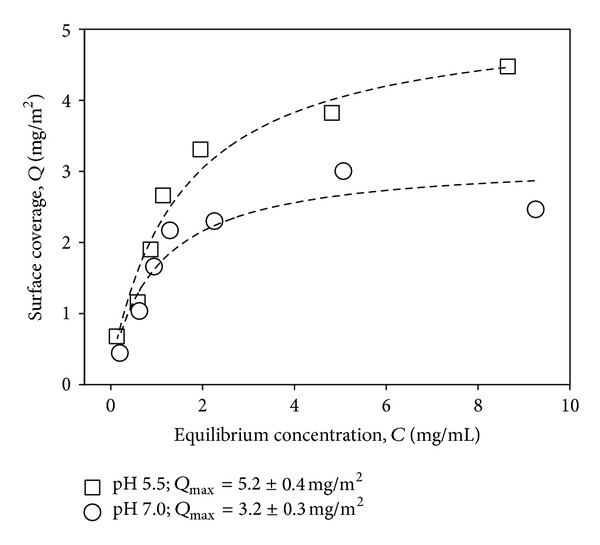
BSA adsorption isotherms on AMS. Adsorption was carried out in 10 mM PB at pH 5.5 and pH 7.0. The amount of BSA adsorbed on AMS was plotted against the equilibrium BSA concentration, and the curves were fitted with Langmuir equation: *Q* = *Q*_max⁡_ *K*_*l*_ *C*/(1 + *K*_*l*_ *C*), *Q* = surface coverage by protein at equilibrium [mg/m^2^], *K*_*l*_ = Langmuir constant [mL/mg], *C* = equilibrium concentration [mg/mL], and *Q*_max⁡_ = maximal surface coverage at equilibrium [mg/m^2^].

AMS had a specific surface area of 518 m^2^/g (Table 1). The majority of this surface area is present inside AMS particles and represents the walls of the micropores. External surface area was 15 m^2^/g. Molecular size of BSA [42] is 4 × 4 × 14 nm^3^. The protein molecules were assumed not to be adsorbed inside the micropores of AMS considering its narrow pore diameter of 0.5 nm [26]. The repeated experiments showed similar adsorption isotherms. Only one set of data is presented in this paper. According to the adsorption isotherms of BSA on AMS at two experimental pH conditions (Figure 2), the equilibrium surface coverage (*Q*_max⁡_) for BSA was higher at pH 5.5 (5.2 ± 0.4 mg/m^2^) than at pH 7.0 (3.2 ± 0.3 mg/m^2^). The Langmuir constants (*K*_*l*_) were 0.7 ± 0.1 mL/mg and 1.0 ± 0.4 mL/mg at pH 5.5 and 7.0, respectively. Considering the standard error of fitting, the Langmuir constant does not seem to be pH dependent.

### 4.2. Protein Release

**Figure 3:**
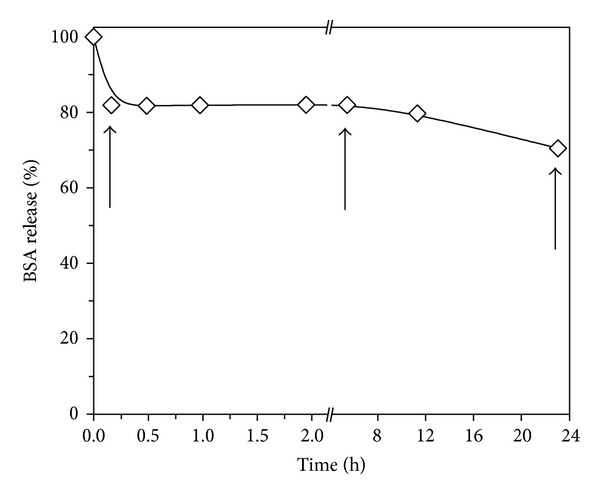
*In vitro* cumulative release of BSA from AMS in 10 mM PB at pH 5.5. The amount of BSA released was calculated as the % of BSA initially present on AMS. For this experiment, 100 mg AMS loaded with 7.8 mg (or 5.2 ± 0.4 mg/m^2^ available surface area) BSA was introduced in 5 mL PB. The amount of BSA adsorbed on AMS was calculated using the equilibrium surface coverage (*Q*_max⁡_ from Figure 2) and the surface area available for protein adsorption (Table 1). The arrows indicate the samples that were characterized with N_2_ physisorption after the release experiment.

Similar release profiles were observed for the repeated experiments. Only one set of data is presented in this paper. AMS adsorbed more BSA at pH 5.5 compared to pH 7.0 (Figure 2). Therefore, pH 5.5 was selected for loading AMS samples with BSA for the release experiments. 100 mg of AMS loaded with 7.8 mg of BSA (5.2 ± 0.4 mg/m^2^) was introduced into release medium (5 mL of 10 mM PB, pH 5.5). BSA release profile (Figure 3) over a period of 24 h depicts three distinguished regions. There was an immediate (burst) release of ca. 18% of adsorbed BSA (first data point; after 10 min). Subsequently, until 6 h, no additional protein was released. Later than 6 h, further slow BSA release was observed for the remainder of the experiment. After 24 h, ca. 30% BSA was released from AMS, and the remaining protein was still present on the surface.

### 4.3. N_2_ Physisorption

**Figure 4:**
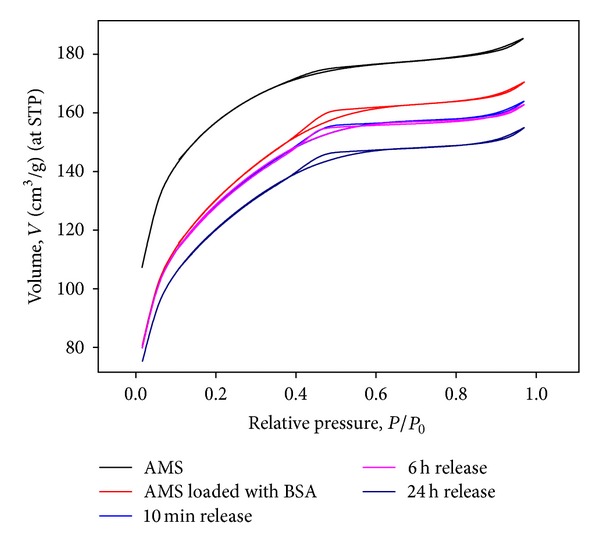
N_2_ adsorption/desorption isotherms of AMS carrier and recovered AMS sample after release of BSA for 10 min, 6 h, and 24 h. Residual protein was removed by calcinations at 400°C. N_2_ isotherms for AMS after 10 min BSA release coincide with 6 h curve.

 All AMS samples showed strong N_2_ uptake at low relative pressure of N_2_ which is typical for a microporous material (Figure 4). After BSA loading followed by removal of BSA, AMS showed a decrease of surface area from 518 to 448 m^2^/g (Table 1). Apparently, the protein adsorption procedure causes some damage to AMS. BSA-loaded samples incubated in release medium for 10 min and 6 h had a same surface area and porosity as after the adsorption experiment. In the first 6 h of the release, microporous structure of AMS was not further altered. After 24 h of protein release, however, the surface area and the microporosity were further decreased (Table 1; Figure 4).

### 4.4. Scanning Electron Microscopy

**Figure 5:**
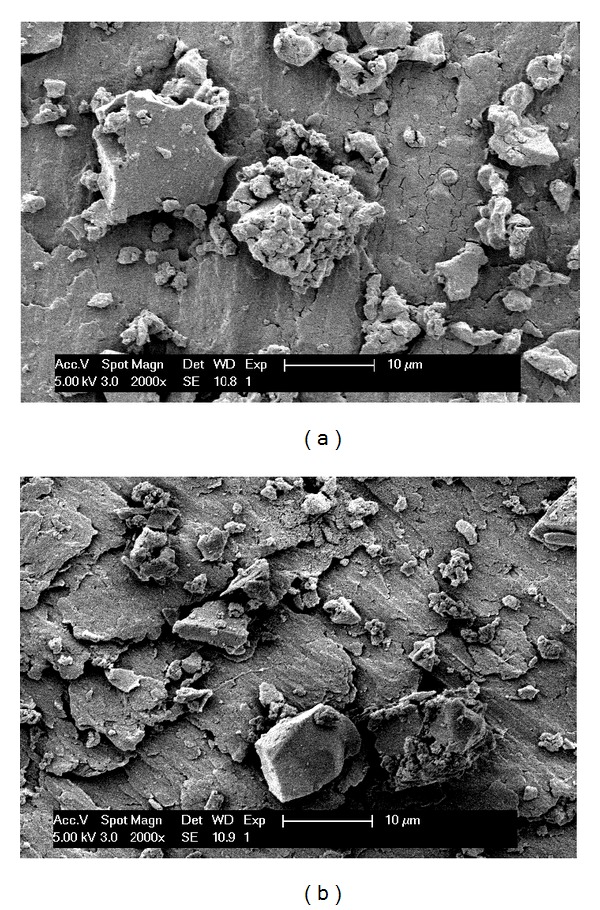
SEM images of AMS particles (a) before and (b) after BSA adsorption.

 SEM images of AMS particles (Figure 5) before and after adsorption of BSA do not show visible degradation of the surface. 

### 4.5. *ζ* Potential Measurement

 Both AMS and BSA had a negative *ζ* potential. Isoelectric point (pH(I)) of BSA [43] is at pH 5.0. The *ζ* potential of AMS was significantly lower at pH 7.0 (−29 mV) than at pH 5.5 (−15 mV). This is a common behavior of silica materials. Negative surface charge of AMS is due to the acid dissociation of silanol groups. The pH(I) of silica is around pH 2. The higher the pH is, more negative is the *ζ* potential.

### 4.6. *In Vivo* BSA Release Profile

**Figure 6:**
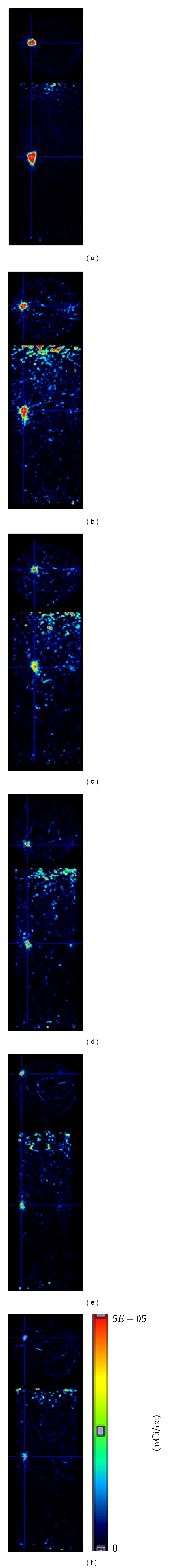
*μ*-PET images at time points (a) 0 h, (b) 4 h, (c) 24 h, (d) 48 h, (e) 72 h, and (f) 96 h. These images show transverse slices (perpendicular to the tibiae) in the upper half of the images and coronal slices (longitudinal to the tibiae) in the lower half of image. Color bar indicates relative intensities for ^124^I.

**Figure 7:**
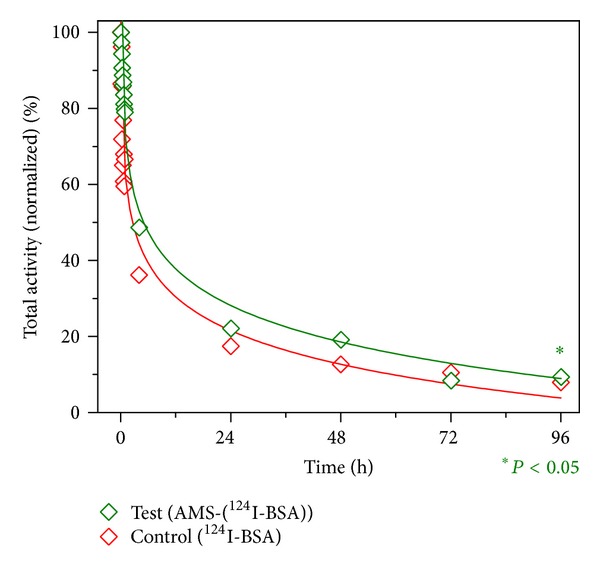
Total activity of ^124^I represents the amount of BSA retained in the bone marrow cavity. Data points are fitted with a logarithmic function, and *R* ^2^ values for the test and control legs are 0.97, and 0.95 respectively. The difference in the 2 profiles is statistically significant (*P* < 0.05).

 The decrease in the activity of ^124^I was considered to be the result of a decrease of radioactivity in time as well as the exit of the protein from the bone marrow cavity through the vascularization.  Figure 6 shows transverse sections (perpendicular to the tibial axis) and coronal sections (parallel to the axes of the tibiae) at various time points. The transverse sections were used for the measurement of  ^124^I activity. Retention profiles of ^124^I-BSA in the 2 legs of the animal with (test) or without (control) AMS are shown in Figure 7. A burst release of ^124^I-BSA could be observed for both measurements in the initial few hours of the experiment. The statistical analysis indicates a significant difference (*P* < 0.05) between the two profiles.

## 5. Discussion 

 Protein adsorption on a solid surface is a complex phenomenon. Literature reveals quite a variation in equilibrium surface coverage values for silica adsorbent systems [44–46]. There are also debates regarding the adsorption mechanism of proteins (monolayer versus multilayer adsorption) and on an appropriate experimental design for studying protein adsorption depending on whether equilibrium or kinetic information is aimed at [47]. But there is little doubt about the influence of physicochemical properties of the surface (such as electric charge, presence and type of functional groups, surface roughness, and porous structure), the protein structure, and the nature of the solvent on protein adsorption [48]. Protein adsorption on a substrate is governed to a large extent by electrostatic interactions and by electrical charge on protein molecules and substrate [45]. In aqueous environment, protein molecules are polyampholytic polymers, carrying both positive and negative charges. At any pH condition, due to the presence of both types of charges on the protein molecule, the charge on the substrate will eventually find a balancing charge on the protein molecule provided that the protein molecule is flexible enough to adapt a required structure for adsorption [49]. Therefore, flexible protein molecules will adsorb on surfaces irrespective of their net surface charge provided they have enough time for restructuring required for adsorption.

 In the present study, for the adsorption of BSA on AMS, the silica adsorbent as well as the protein had a negative *ζ* potential, showing that both substrate and protein molecules possess a net negative surface charge under applied experimental conditions. Protein adsorption depends on protein/protein and protein/surface electrostatic interactions. It is generally observed that the amount of adsorbed protein at equilibrium is maximal at pH(I) of the protein (i.e., at pH 5.0 for BSA), and this is considered as the best experimental condition for maximum protein adsorption if other experimental conditions (such as solvent, ionic strength, etc.) are maintained [45]. When protein adsorption is carried out at pH close to the pH(I) of the protein, the protein-solvent interactions are reduced, and protein molecules are more readily available at the adsorption site. Similarly, a lateral repulsion between adsorbing protein molecules is suppressed due to their approximate zero net surface charge at pH(I) [47]. In the present study, maximal BSA adsorption was indeed observed when the adsorption was carried out at pH near pH(I) of the protein (i.e., at pH 5.5). According to the *ζ* potential results, AMS surface potential also contributes to an increased adsorption capacity at pH 5.5. *ζ* potential of AMS was significantly less negative at pH 5.5 resulting in less repulsion with BSA molecules and leading to an increased number of adsorption sites on the surface. 

 Considering BSA molecules as rigid spheres with a volume of 224 nm^3^ (derived from the dimensions of 4 × 4 × 14 nm^3^according to its crystal structure) [42], 1.71 mg of protein is needed for monolayer coverage of a flat surface of 1 m^2^. In this estimation, ellipsoidal BSA molecules are assumed to be randomly oriented when adsorbed on AMS surface. Experimentally determined plateau coverage value (*Q*_max⁡_ = 5.2 mg/m^2^ at pH 5.5) was higher than the model value of 1.71 mg/m^2^. In this case *Q*_max⁡_ value was calculated assuming AMS with a surface area of 15 m^2^/g. The *t*-plot model that has been used for estimating external surface area is an approximation and experimental value that might present some error as well, but this will not explain the large difference between experimental and predicted *Q*_max⁡_. High experimental adsorption capacity may indicate that the random orientation of BSA molecules is not an appropriate model and that BSA molecules on AMS surface adopt a closer packing. If ellipsoidal BSA molecules would be adsorbed end-on with longer dimensions perpendicular to the surface, theoretical value for the monolayer surface coverage by BSA is 8.8 mg/m^2^. And for side-on adsorption, this value is 2.5 mg/m^2^. Experimental value of monolayer surface coverage of 5.2 mg/m^2^ is in between the values for end-on and side-on adsorptions. This observation shows that the adsorption capacity is in the range of protein monolayer adsorption. Depending on the model, the presence of a partial second protein layer cannot be ruled out. 

 It is instructive to compare BSA adsorption on AMS with that on other silica materials. Mesoporous (50 nm > pore size > 2 nm) materials have pores that may accommodate protein molecules depending on the exact pore size. Mesoporous silica is expected to provide more surface area for BSA adsorption compared to AMS considering its internal surface area which may also be available for protein adsorption. In this study, the amount of BSA adsorbed on AMS surface at plateau formation is 5.2 mg/m^2^ (at pH 5.5), which is larger than BSA adsorption on other silica surfaces. For example, *Q*_max⁡_ values found out by Ho et al. [44] (pH 4.2) and Kondo and Higashitani [45] (pH 5.5) are 3.15 mg/m^2^ and 3.1 mg/m^2^, respectively. These comparisons suggest that a specific interaction between protein and AMS exists which enhances its protein adsorption capacity. Possibly, side chains of BSA could accommodate themselves either partially or completely into the micropores of AMS, thereby increasing the contact area and van der Waals interaction between protein and surface. 

 Adsorbed protein molecules are released from any surface when subjected to an increase in ionic strength and/or pH change away from its pH(I). Such situation would be encountered in an intended application of implanted surfaces with adsorbed protein [46]. To study the influence of possible surface microstructure changes of AMS on protein release kinetics, the release experiments were carried out with same experimental conditions as used for loading of the protein. Initial burst release of BSA suggests presence of loosely bound protein molecules [47]. Thereafter, there was no further protein release till 6 h showing that the protein-surface attraction was very strong despite of the lower protein concentration in the solution. AMS underwent changes in its microstructure in release medium. Significant reduction of the micropore area and volume in the period from 6 to 24 h protein release may have been responsible for BSA release mechanism during this period. Due to the reduction of the micropore area, the contact area between BSA residues or side chains and AMS surface decreased. Consequently, the protein-surface bond was weakened leading to a release of BSA molecules. Although initial amount of released BSA (burst) was quite high, it was still a minor fraction (ca.18%) of estimated total amount of protein initially present on the surface. So, after this initial burst release, there was enough adsorbed protein present on the surface for further release. Therefore, AMS potentially can be used as a protein delivery vehicle for release over longer period than 24 h. The release is dependent on aging of the silica and the loss of its microporosity. The latter is the result of a release medium-mediated restructuring of silica, known in the literature as Ostwald ripening [50]. In this process, silica in monomeric form (orthosilicic acid, Si(OH)_4_) is released into the solution from smaller particles and gets redeposit on larger ones. This phenomenon may have been responsible for the changes in microporous surface of AMS.

 For any biomaterial to be used as a BMP delivery vehicle, it is crucial to find out the BMP release profile at the bone/implant interface *in vivo* because this affects the effectiveness of the protein in terms of bone regeneration. Once *in vivo* BMP release profile of a delivery vehicle is known, its effect on the bone regeneration can be evaluated so that a direct correlation can be made to the positive effect of bone regeneration if any. In this study, *in vivo* BSA release was determined from particulate AMS instead of a coating to provide large surface area for protein adsorption for this exploratory experiment. BSA was used as a model protein. Due to their larger size (80–125 *μ*m), AMS particles as used in this experiment are confined to the bone marrow cavity and cannot escape through the blood vessels. The protein molecules on the other hand are much smaller and can therefore easily be released from the bone marrow cavity. It should be noted here that the decrease in the activity of ^124^I as measured on the *μ*-PET-scan of the tibia is due to the release of BSA molecules from the bone marrow cavity. As per the design of the experiment, the protein releasing from the cavity may (test) or may not (control) have been adsorbed on the AMS surface. But the differences in the retention profiles suggest the effect of AMS as a barrier to the release of BSA molecules from the test leg. Although ^124^I-BSA retention profiles for the test and the control materials in the animal are similar in pattern, and an exponential decrease can be observed in both cases, statistical analysis shows a significant difference when the two profiles were compared. This experiment supports the hypothesis that AMS supports ^124^I-BSA retention in the bone marrow cavity. Sustained release of the growth factor molecules along with the bulk properties of a delivery vehicle is an important factor for a desired positive tissue response [51]. Despite the statistical difference in the protein retention profiles for the test and the control, experimentation with more test animals is necessary. Moreover, the effect of particular release profile on bone regeneration needs to be checked with BMPs adsorbed on AMS coatings on implants.

 Finally the relevance of the present study in terms of AMS as a potential BMP delivery system needs to be discussed. Released quantity and profile of BMPs may affect different stages of cell differentiation and proliferation during the bone formation lineage [52]. There is no sufficient information available regarding BMP release kinetics necessary for required tissue regeneration [24]. Therefore, to gain basic knowledge about the effect of particular protein release profile on the process of osseointegration, it is necessary to find out new biocompatible delivery vehicles which are either inert or do not adversely affect the process of osseointegration and can deliver BMPs with a possibility of different release profiles. The present study has explored and confirmed the potential use of AMS as a suitable material for protein delivery. In addition, it may be anticipated that synthetic control of the AMS structure presents a significant advantage for protein release applications. By changing synthesis parameters, AMS micropore size can be controlled, which in turn can control the protein adsorption by providing different micropore areas for amino acid side chains of the proteins. Moreover this study demonstrated higher adsorption of BSA on AMS in adverse conditions such as negative surface charge on both adsorbent and adsorbate. BMPs are expected to interact with AMS at least as strong as BSA because it has its pH(I) in the basic region. With more amount of BMP adsorbed on AMS surface, the protein release is expected for a longer period. However, further investigation is necessary to decide the fate of AMS as a delivery vehicle for BMPs. 

## 6. Conclusions

 On microporous silica material with a pore size smaller than the size of the protein such as AMS, strong BSA adsorption was observed. Providing a silica adsorbent with micropores was shown to be helpful for slowing down BSA release. AMS shows some aging in an aqueous environment and gradually loses its microporosity provoking release of BSA molecules into aqueous medium. Tuning the microporosity and foreseeing a degradation of microporous structure are tools for designing protein release kinetics from AMS surfaces. In an exploratory experiment it was observed that the *in vivo* monitoring of protein release/retention by means of *μ*-PET is possible and can readily provide quantitative data. This investigation indicates the potential of AMS as a delivery vehicle for protein molecules. The present results hint at a possible use of AMS coatings in clinical applications as a protein delivery vehicle. Specifically for BMPs, adsorption at neutral pH may increase the protein's concentration in adsorbed state which may aid its delivery for longer periods. 

## Figures and Tables

**Table 1:** Specific surface area of AMS determined from N_2_ physisorption isotherms.

	Micropore volume^a^ (mm^3^/g)	External surface area^a^ (m^2^/g)	BET surface area (m^2^/g)
AMS	260	15	518
BSA loaded	240	15	448
10 min release^b^	230	13	440
6 h release^b^	230	12	441
24 h release^b^	220	13	404

^a^Determined using the *t*-plot method; ^b^after elimination of the adsorbed BSA by calcination.
